# Oxalate Alters Cellular Bioenergetics, Redox Homeostasis, Antibacterial Response, and Immune Response in Macrophages

**DOI:** 10.3389/fimmu.2021.694865

**Published:** 2021-10-21

**Authors:** Parveen Kumar, Kanchan Saini, Vikram Saini, Tanecia Mitchell

**Affiliations:** ^1^ Department of Urology, University of Alabama at Birmingham, Birmingham, AL, United States; ^2^ Laboratory of Infection Biology and Translational Research, Department of Biotechnology, All India Institute of Medical Sciences, New Delhi, India

**Keywords:** oxalate, macrophage, redox homeostasis, cellular bioenergetics, inflammation, kidney stone, *Escherichia coli*

## Abstract

Individuals with calcium oxalate (CaOx) kidney stones can have secondarily infected calculi which may play a role in the development of recurrent urinary tract infection (UTI). Uropathogenic *Escherichia coli* (UPEC) is the most common causative pathogen of UTIs. Macrophages play a critical role in host immune defense against bacterial infections. Our previous study demonstrated that oxalate, an important component of the most common type of kidney stone, impairs monocyte cellular bioenergetics and redox homeostasis. The objective of this study was to investigate whether oxalate compromises macrophage metabolism, redox status, anti-bacterial response, and immune response. Monocytes (THP-1, a human monocytic cell line) were exposed to sodium oxalate (soluble oxalate; 50 µM) for 48 hours prior to being differentiated into macrophages. Macrophages were subsequently exposed to calcium oxalate crystals (50 µM) for 48 hours followed by UPEC (MOI 1:2 or 1:5) for 2 hours. Peritoneal macrophages and bone marrow-derived macrophages (BMDM) from C57BL/6 mice were also exposed to oxalate. THP-1 macrophages treated with oxalate had decreased cellular bioenergetics, mitochondrial complex I and IV activity, and ATP levels compared to control cells. In addition, these cells had a significant increase in mitochondrial and total reactive oxygen species levels, mitochondrial gene expression, and pro-inflammatory cytokine (i.e. Interleukin-1β, IL-1β and Interleukin-6, IL-6) mRNA levels and secretion. In contrast, oxalate significantly decreased the mRNA levels and secretion of the anti-inflammatory cytokine, Interleukin-10 (IL-10). Further, oxalate increased the bacterial burden of primary macrophages. Our findings demonstrate that oxalate compromises macrophage metabolism, redox homeostasis, and cytokine signaling leading to a reduction in anti-bacterial response and increased infection. These data highlight a novel role of oxalate on macrophage function.

## Introduction

The prevalence and recurrence of kidney stones in children and adults are increasing worldwide (1, 2). The economic burden from kidney stone disease is several billion dollars annually and is predicted to rise due to increasing stone prevalence (1, 3). The multifactorial etiology of the disease is substantiated by several risk factors such as diet, intestinal micro-flora composition, genetics, metabolic conditions, and geographical location which all contribute to the development and progression of stone formation (4–8). In addition, patients with kidney stones have a heightened risk for end-stage renal failure (9), chronic kidney disease (10), diabetes (11), and cardiovascular disease (12). There have been reports of an association between kidney stone formation and urinary tract infection (UTI) in certain patient cohorts (13–17).

*Escherichia coli* (*E. coli*) is the most common pathogen causing UTI (18, 19). *E. coli* has been shown to aggregate around calcium oxalate (CaOx) crystals (15, 20), the precursors of the most common type of kidney stone, CaOx. In addition, *E. coli* has been isolated from some individuals harboring CaOx stones (14). Increased consumption of oxalate rich foods may lead to elevated urinary oxalate levels in patients with CaOx stones which have been found to be associated with recurrent UTIs (4, 21–22). It has been reported that female kidney stone patients with recurrent UTIs have significantly higher urinary oxalate excretion compared to patients without UTIs (23). In addition, removal of stones from patients with recurrent UTIs significantly reduces the risk of subsequent UTI (24, 25). These reports suggest a possible association between urinary oxalate and uropathogenic *Escherichia coli* (UPEC) infection susceptibility.

We have previously reported that patients with CaOx kidney stones have suppressed monocyte cellular energetics compared to healthy subjects (26). We also determined that soluble oxalate compromises cellular bioenergetics in THP-1 monocytes (27). These observations were corroborated recently in human studies where we reported that healthy adults who consumed an oxalate enriched meal had decreased monocyte cellular bioenergetics and mitochondrial complex I activity, suggesting dietary oxalate could influence monocyte function in certain individuals (28). Oxalate has been shown to cause monocytes to differentiate into pro-inflammatory macrophages rather than anti-inflammatory macrophages (29).

Considering the multi-faceted impact of oxalate on monocytes, we hypothesized that oxalate compromises the metabolic health of macrophages and their ability to respond to bacterial infection due to reduced immune response. We developed an *in vitro* model where macrophages were exposed to oxalate over an extended time frame to simulate the effect of an oxalate-enriched diet on macrophages. Herein, we report that oxalate exposure alters macrophage cellular bioenergetics, mitochondrial complex activity, ATP levels, redox homeostasis, and mitochondrial gene expression. Oxalate treated macrophages also exhibited a compromised anti-bacterial response which may be due to disrupted inflammatory cytokine secretion.

## Materials and Methods

### Reagents

The following reagents were purchased from Sigma Aldrich (St. Louis, MO): antimycin A, ADP, ascorbate, azide, FCCP (carbonyl cyanide 4-(trifluoromethoxy) phenylhydrazone), malate, oligomycin, pyruvate, rotenone, succinate, and TMPD (N,N,N′,N′-Tetramethyl-p-phenylenediamine). All other reagents purchased are noted elsewhere.

### Bacterial Strain and Culture Condition

Uropathogenic *Escherichia coli* CFT073, an acute pyelonephritis strain (ATCC # 700928) was used for these studies. CFT073 was cultured on solid liquid broth (LB) agar or in liquid LB media at 37°C for 48 hrs under static condition (30). CFT073 was characterized for their growth and colony-forming units (CFU) were enumerated before initiating experiments. CFT073 was cultured overnight in LB media and the OD600 was determined to calculate multiplicity of infection (MOI) for all experiments. Bacteria were harvested in phosphate buffered saline (PBS) and adjusted to 1x10^6^ CFU/ml.

### Cell Culture Model

THP-1 cells (a human monocytic derived cell line) were grown and maintained in RPMI 1640 medium supplemented with 10% fetal bovine serum and 2-mercaptoethanol at 37^°^C in a CO_2_ incubator. THP-1 cells (passage 3-6) were exposed to sodium oxalate (NaOx; 50 µM) for 48 hrs prior to being differentiated into macrophages for 48 hrs using 200 nM phorbol 12-myristate 13-acetate (PMA). THP-1 cells were exposed to oxalate to mimic oxalate exposure to circulating monocytes prior to differentiation. Cells were subsequently washed and allowed to rest for 24 hrs before being exposed to CaOx crystals (50 µM) for 24 hrs to mimic *in vivo* conditions within the kidney. In additional experiments, macrophages were subsequently exposed to CFT073 (MOI 1:2 unless noted otherwise) for 1 hr. Cells were then treated with media containing antibiotics (gentamycin, 200 µg/ml) for 1 hr to remove extracellular bacteria. Antibiotic containing media was removed and cells were washed with fresh antibiotic free media prior to performing additional experiments.

### Cell Viability Assay

Cell viability was assessed using a CCK8 assay kit (Cat. #CK04-05; Dojindo Molecular Technologies, Inc., Rockville, MD) according to the manufacturer’s instructions. The CCK8 assay is based on the reduction of tetrazolium salt WST-8 by cellular dehydrogenases and results in the production of a yellow-colored formazan dye which is directly proportional to the number of viable cells. The cellular media was removed from cells following treatment as described above and replaced with fresh media containing WST-8 for 2 hours. Subsequently, absorbance was measured at 450 nm using a plate reader.

### Cellular Bioenergetics and Mitochondrial Complex Activity Analyses

Cellular bioenergetics and mitochondrial complex activities were assessed using the Seahorse Analyzer (Agilent Technologies, Santa Clara, CA). THP-1 cells were treated with NaOx for 48 hrs prior to being plated (25,000 cells per well) in XF96 well plates and differentiated into macrophages using PMA. Macrophages were allowed to rest 24 hours before being treated with CaOx crystals (50 µM) for 24 hours. Macrophages were subsequently exposed to uropathogenic *E. coli* (MOI 1:2) for 1 hour followed by antibiotic treatment (gentamycin, 200 µg/ml) for 1 hour to remove extracellular bacteria. Cells were washed with XF media and allowed to equilibrate prior to performing the mitochondrial stress test, which consisted of sequentially exposing macrophages to oligomycin (1.5 µg/ml), FCCP (2 µM), and Antimycin A (10 µM) to determine the oxygen consumption rate (OCR) over time (28). Additionally, the glycolytic stress test (GST) was performed to assess the extracellular acidification rate (ECAR) by exposing cells to glucose (10 mM), oligomycin (1 µg/ml), and 2-deoxyglucose (50 mM). In additional experiments, cells were permeabilized with the XF Plasma Membrane permeabilizer (Agilent Technologies, Santa Clara, CA) before being exposed to different substrates to assess mitochondrial complex activity (31). All data were normalized to cellular protein content.

### ATP Analysis

Intracellular ATP content of macrophages was determined using Enliten ATP assay system bioluminescence detection kit (Cat. # FF2000; Promega, Madison, WI). Following treatment, macrophages were washed with 1X PBS and lysed using 1% tricholoro acetate (TCA) – 1X Tris-EDTA buffer (pH 7.5). Cell lysates were diluted 10 times using 1X TE buffer followed by centrifugation at 13000 rpm for 10 min. The supernatant was used to measure ATP content. Each sample (100 µl) was mixed with the reagent (100 µl) before immediately measuring luminescence on a SpectraMax L Microplate Reader (Molecular Devices, San Jose, CA). The ATP concentration in cells was determined using an ATP standard curve. All samples and standards were analyzed in duplicates.

### Reactive Oxygen Species (ROS) and Mitochondrial Membrane Potential Assays

Relative total cellular ROS and mitochondrial superoxide levels were evaluated in macrophages using H2DCFDA (Cat. # D399, ThermoFisher Scientific, Watham, MA) and MitoSOX Red mitochondrial superoxide (Cat. # M36008, ThermoFisher Scientific, Watham, MA) fluorescence dyes, respectively. Macrophages were plated in 96-well plates (25,000 cells/well) and treated as described above. In some experiments, cells were treated with the positive control menadione (100 μM) for 1 hour before measuring total ROS. Following treatment, cells were washed twice with HBSS or PBS before being stained with H2DCFDA (5 µM in PBS, 15 mins) or MitoSOX Red (5 µM in HBSS, 15 mins). Cells were washed three times with HBSS or PBS prior to measuring fluorescence signal using a Synergy HT microplate reader (BioTek, Winooski, VT).

Hydrogen peroxide (H_2_O_2_) levels were measured using the Amplex Red Hydrogen Peroxide/Peroxidase assay kit (Cat. #A22188, Invitrogen). In brief, cells were washed with Krebs-Ringer phosphate buffer (KRPG) before adding 100 µl of reaction mixture (containing 50 µM Amplex Red reagent and 0.1 U/ml HRP in KRPG) to each well. Cells were incubated for 1 hr at 37°C and relative fluorescence units were measured at 560/590 (Ex/Em) using a Synergy HT microplate reader (BioTek, Winooski, VT). NADPH levels were measured using a NADP/NAPDH quantitation colorimetric kit (Cat. #K347, BioVision, Milpitas, CA) according to the manufacturer’s instructions. Following treatment, cells were washed once with 1X PBS and subsequently incubated with 400 µl NADPH extraction buffer on ice for 10 mins. All cellular content was collected and centrifuged at 21,000 g for 10 min at 4°C. The supernatant was collected and 200 µl was incubated in a water bath (60°C) for 30 min. Samples were immediately cooled on ice before adding 50 µl of sample to a 96-well plate along with NAPDH standards (0–100 pmol/well). The NADP cycling mix and NADP enzyme mix was added to each well and incubated for 5 min at RT. Next, the NADPH developer was added to each well for 2 hrs at RT. The concentration of NADPH was determined by reading all samples and standards at 450 nm.

Mitochondrial membrane potential was evaluated in macrophages using Tetramethylrhodamine ethyl ester, perchlorate (TMRE). Cells were incubated for 30 min with TMRE and fluorescence was observed at 549/575 nm using a plate reader. In additional experiments, cells were incubated simultaneously with MitoTracker Red and MitoTracker Green for 30 min before measuring fluorescence at 490/516 and 579/599, respectively using a plate reader.

### Quantitative Real Time–PCR (qRT-PCR)

RNA was isolated from macrophages using a RNA easy mini kit (Cat. #74104; Qiagen, Germantown, MD). 1 µg RNA was used for the synthesis of cDNA using the Quantitect Reverse Transcription kit (Cat. #205311; Qiagen, Hilden, Germany). Primers were designed using Primer3 (32) and synthesized from IDT Technologies (Coralville, IA) (**Table 1**). qRT-PCR was performed using Applied Biosciences master mix (Cat. #A25742) and platform. Quantification was done by ΔΔCt method using GAPDH as reference gene and untreated cells as controls (33, 34).

**Table 1:** List of primers used for real time PCR.

Gene name	Forward primer	Reverse primer
GAPDH	5’-CTCCTGTTCGACAGTCAGCC-3’	5’-TGGAATTTGCCATGGGTGGA-3’
VDAC1	5’-GGGTGCTCTGGTGCTAGG T-3’	5’-GACAGCGGTCTCCAACTTCT-3’
NDUFS7	5’-CGCAAGGTCTACGACCAGAT-3’	5’-CTCACCACCGAGTAGGAATAGTG-3’
ND1	5’-CTACTACAACCCTTCGCTGAC-3’	5’-GGATTGAGTAAACGGCTAGGC-3’
IL-1β	5’-CAATGGTTGCTGTCTCATCAGC-3’	5’-CTAGGCCACAACAACTGACG-3’
TFAM	5’-GATGCTTATAGGGCGGAG-3’	5’-GCTGAACGAGGTCTTTTTGG-3’
PGC1α	5’-CCTGTGGATGAAGACGGATT-3’	5’-TAGCTGAGTGTTGGCTGGTG-3’
IL-6	5’-TTCCAAAGATGTAGCCGCCC-3’	5’-CACCAGGCAAGTCTCCTCAT-3’
IL-10	5’-AGAACCTGAAGACCCTCAGGC-3’	5’-CCACGGCCTTGCTCTTGTT-3’

### Western Blotting

IL-6 and Il-1β levels were determined in the supernatant by western blotting. The supernatant was collected after treatment and protein was precipitated using 4 volumes of ice-cold acetone. The protein was dissolved in RIPA buffer containing protease inhibitors before determining the concentration of cellular protein using the Pierce Coomassie Bradford protein assay (Thermo Fisher Scientific, Cat. #23236). Samples were separated on a 4-15% gel before being transferred to a PVDF membrane to probe for IL-1β (1:500), IL-6 (1:500), and β-actin (1:2000) as previously described (27). Blots were incubated with primary antibodies overnight at 4°C before being treated the following day with anti-rabbit secondary horse radish peroxidase-conjugated antibodies (1:10,000 for 1 hr at room temperature; Abcam, Cambridge, UK). Membranes were washed, incubated with a Luminata Forte Chemiluminescence (Millipore) for protein detection, and analyzed using an ImageQuant LAS 4000 imager and software (GE Healthcare Life Sciences, Marlborough, MA).

### Evaluation of Intracellular Colony Forming Units (CFU) in THP-1 Derived Macrophages

The intracellular CFU count was evaluated to determine the anti-bacterial response of THP-1 macrophages. Macrophages were treated with oxalate as described earlier and subsequently challenged with CFT073 (MOI 1:5) for 1 hr. Cells were treated with media containing antibiotic (gentamycin, 200 µg/ml) for 1 hr to eliminate extracellular bacteria and thereafter incubated for an additional 2 and 6 hrs in antibiotic (gentamycin, 20 µg/ml) containing media (35). At each time-point post-exposure (2, 4, and 8 hrs), the cells were washed with 1X PBS and lysed in 0.025% SDS. Serial dilutions of the lysates were plated on LB agar plates and incubated at 37°C for 12-16 hrs to calculate the relative differences in CFU/ml. In additional experiments, the bacterial count in the cellular media was determined by serially diluting the media on LB agar plates.

### Evaluation of CFU in Primary Macrophages

Peritoneal and bone marrow derived macrophages (BMDM) were isolated from male C57BL/6J mice (8 to 12 weeks old). Mice were maintained and housed at the Central Animal Facility, All India Institute of Medical Sciences (AIIMS), New Delhi under pathogen free conditions with *ad libitum* access to water and standard rodent chow diet. All animal procedures were performed as per the approved protocols from the Institutional Animal Ethics Committee, AIIMS, New Delhi (Project id: N1125).

Briefly, peritoneal macrophages were collected from the peritoneal cavity as previously indicated (36). Red blood cells were removed from macrophages using ACK lysing buffer followed by washing with PBS. Cells were cultured and grown in RPMI 1640 with 10% fetal bovine serum and penicillin-streptomycin (100 U/mL penicillin, 100 μg/mL streptomycin) at 37°C in a CO_2_ incubator for 3 days. Likewise, BMDMs were isolated from the femurs of male C57BL/6J mice. BMDM were collected from the bone marrow of the dissected femur by gently expelling the bone marrow cavity with 1x PBS (37). Cells were washed with 1x PBS and cultured in DMEM media with 10% FBS, penicillin-streptomycin (100 U/mL penicillin, 100 μg/mL streptomycin) and M-CSF (10 ng/mL) at 37^°^C in a CO_2_ incubator (36). At Day 3 and 6, the media was removed and re-supplemented with fresh DMEM containing 10% FBS, penicillin-streptomycin (100 U/mL penicillin, 100 μg/mL streptomycin) and M-CSF (10 ng/mL). After 9 days, the differentiated cells were used for experiments.

For infection assays, both peritoneal and BMDM macrophages were seeded (5 x 10^4^ cells/well) in a 24-well plate in their respective media and allowed to rest for 24 hrs at 37°C in a CO_2_ incubator. Cells were independently exposed to either calcium oxalate (CaOx; 50 μM) or sodium oxalate (NaOx; 50 μM) for 72 hrs. Following exposure, macrophages were washed and infected with CFT073 (MOI 1:5) as described earlier. Cells were treated with media containing antibiotic (gentamycin, 200 µg/ml) for 1 hr to eliminate extracellular bacteria. Subsequently, cells were washed twice with 1x PBS and re-supplemented with antibiotic free media (considered as T=0). For CFU enumeration, cells were lysed with 0.025% SDS at time points 0, 4, and 8 hrs post-infection. Cell lysates were serially diluted and plated on LB agar plates. Plates were incubated at 37^°^C for 16 hrs followed by CFU enumeration.

### Phagocytosis Assay

BMDMs were seeded in a 6-well plate (10^6^ cells/well) and allowed to rest for 24 hrs at 37°C in a CO_2_ incubator. Cells were then exposed to calcium oxalate (CaOx; 50 μM) or sodium oxalate (NaOx; 50 μM) for 72 hrs. Subsequently, macrophages were washed and fluorescent latex beads (Cat. #15702-10 Fluoresbrite^®^ Microparticles, Polysciences) were added to the wells (100 beads/cell reconstituted in 1x PBS) (38, 39). Afterwards, cells were kept at 37°C for 30 minutes while shaking. Cells were washed twice with 1x PBS and visualized using a Cytation 1 Cell Imaging Multimode reader (BioTek) at 488/510 nm.

### Cytokine Assays

Inflammatory cytokine levels were measured using ELISA Max™ human cytokine Interleukin-6 (IL-6) and Interleukin-10 (IL-10) kits (Cat. # 430504 & 430601, BioLegend, San Diego, CA, USA) and IL-1β human ELISA kit (Cat. # BMS224-2, Invitrogen, Carlsbad, CA, USA) according to the manufacturer’s instructions. The media supernatants were collected and spun to remove any cellular debris prior to saving supernatants in a -20°C freezer. Samples were thawed and diluted in sample diluent (1:2) prior to analysis.

### Statistical Analyses

All statistical analyses were performed using GraphPad Prism software, version 9.1.1 (La Jolla, CA). Data are expressed as mean ± standard error of the mean using n=3 or more determinations. Multiple comparisons were analyzed using one-way analysis of variance with Tukey’s *post hoc* test. A p value <0.05 was considered statistically significant.

## Results

### Oxalate Reduces Macrophage Cellular Bioenergetics, Mitochondrial Complex Activity, and ATP Production in Macrophages

**Figure 1:**
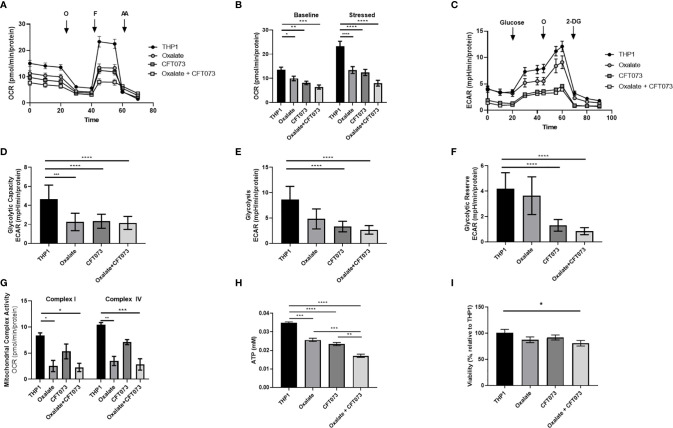
Oxalate reduces cellular bioenergetics and ATP production in THP-1 macrophages. THP-1 macrophages were plated in Seahorse plates prior to being exposed to oxalate with or without CFT073 (MOI 1:2) as described in the *Materials and Methods* section. The Seahorse XF Analyzer and **(A)** The “Mitochondrial Stress Test” was used to assess macrophage oxygen consumption rate over time. **(B)** Baseline and stressed OCR parameters were calculated from the “Mitochondrial Stress Test”. **(C)** The “Glycolysis Stress Test” was used to assess macrophage extracellular acidification rate over time. **(D)** Glycolytic capacity, **(E)** glycolysis, and **(F)** glycolytic reserve parameters were derived from the “Glycolysis Stress Test”. **(G)** Mitochondrial Complex I and IV activities were assessed after permeabilizing the cells and adding substrates as described in the methods. **(H)** Intracellular ATP levels in macrophages were determined as described in detail in the *Materials and Methods* section. **(I)** Viability of THP-1 macrophages using the CCK8 assay. All experiments were performed at least three times with n=5-8 replicates. Data are expressed as means ± SEM. *p < 0.05, **p < 0.01, ***p < 0.001, ****p < 0.0001 is significant compared to control THP-1 macrophages or CFT073 alone.

An assessment of cellular bioenergetics is comprised of processes that are critical for the metabolic health of cells. To determine the impact of oxalate on cellular bioenergetics, we assessed macrophages treated with and without CFT073. We determined cells exposed to oxalate and CFT073 have reduced OCR compared to control macrophages (**Figure 1A**). OCR was further reduced when macrophages were exposed to both oxalate and CFT073 (**Figure 1A**). As shown in **Figure 1B**, the OCR was significantly reduced in all treatment groups under baseline and stressed (maximal OCR) conditions. To further characterize the effect of oxalate on energy production, we performed the glycolysis stress test (**Figure 1C**). The ECAR was impaired in macrophages exposed to oxalate with or without CFT073 infection compared to THP-1 control cells (**Figure 1C**). The glycolytic capacity, which is a measure of the maximum capacity of glucose converting to pyruvate or lactate, was significantly decreased in all treatment groups (**Figure 1D**). Glycolysis and the glycolytic reserve (the difference between glycolytic capacity and glycolysis) were not significantly different between control macrophages and cells exposed to oxalate alone (**Figures 1E, F**). However, both glycolysis and the glycolytic reserve were significantly reduced in macrophages exposed to CFT073 with or without oxalate (**Figures 1E, F**).

To explore the basis of reduced bioenergetics following oxalate treatment, we assessed mitochondrial Complex I and IV activity. We determined these cells have a significant reduction in Complex I and IV activity with or without CFT073 infection (**Figure 1G**). With a decline in mitochondrial complex activities and an apparent inability of oxalate-treated cells to shift to glycolysis, we posited this phenotype may cause a reduction in ATP and compromise their ability to respond to infection. Consistent with these hypotheses, oxalate caused a significant decrease in ATP levels compared to control macrophages (**Figure 1H**). ATP levels were further reduced when macrophages were exposed to oxalate and CFT073 (**Figure 1H**). A reduction in cellular ATP could be due to cell death. However, oxalate treatment did not impact macrophage viability (**Figure 1I**). In contrast, macrophages treated with oxalate and CFT073 had a significant decrease in viability compared to control cells (**Figure 1I**).

### Oxalate Disrupts Redox Homeostasis in Macrophages

**Figure 2:**
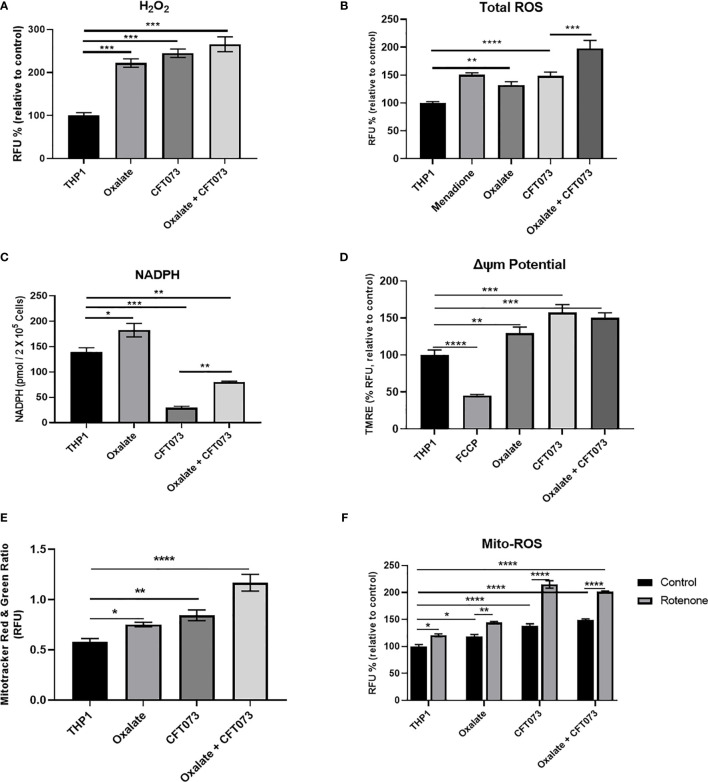
Oxalate increases reactive oxygen species (ROS) levels and mitochondrial membrane potential in THP-1 macrophages. THP-1 macrophages were exposed to oxalate with or without CFT073 (MOI 1:2) prior to assessing **(A)** Hydrogen peroxide (H_2_O_2_; Amplex Red Assay), **(B)** Total ROS (H2DCFDA, fluorescence dye; Menadione (100 μM)-positive control), and **(C)** NAPDH levels. **(D)** Mitochondrial membrane potential was examined using the fluorescence dye, TMRE. FCCP (50 nM) was included as a control. **(E)** MitoTracker Red/MitoTracker Green fluorescence intensity ratio in macrophages. **(F)** Mitochondrial ROS (superoxide) levels were determined following treatment with or without rotenone (3 µg/ml). All experiments were performed at least three times with n=5 replicates. Data are expressed as mean ± SEM. *p < 0.05, **p < 0.01, ***p < 0.001, ****p < 0.0001 is significant compared to control THP-1 macrophages or CFT073 alone.

Mitochondria are a major source of ROS, which are critical determinants of the antibacterial response in immune cells (40). Thus, we evaluated whether compromised cellular bioenergetics and reduced mitochondrial complex activity contributes to elevated ROS production in macrophages. We determined that both H_2_O_2_ and total ROS levels were significantly elevated in macrophages following oxalate exposure (**Figures 2A, B**, respectively). These levels were further exacerbated when cells were treated with CFT073 alone or oxalate. NADPH levels were also significantly elevated following oxalate exposure (**Figure 2C**). However, CFT073 with or without oxalate caused a significant reduction in NADPH levels (**Figure 2C**). We subsequently determined mitochondrial membrane potential was significantly elevated in oxalate treated macrophages in the absence and presence of CFT073 (**Figure 2D**). These observations were further supported by Mito-Tracker Red: Mito-Tracker Green staining in macrophages. As shown in **Figure 2E**, macrophages exposed to oxalate, CFT073, or oxalate with CFT073 had a significant increase in the ratio of Mito-Tracker Red: Mito-Tracker Green staining compared to control cells, suggesting that oxalate can induce mitochondrial hyperpolarization in macrophages. Lastly, we assessed mitochondrial superoxide levels and determined that oxalate treatment significantly increased mitochondrial superoxide levels in macrophages; this was further exacerbated when macrophages were exposed to infection alone or with oxalate and CFT073 (**Figure 2F**). These findings suggested the possibility that reverse electron transport (RET) may be the source for elevated ROS. However, inhibition of Complex I (a major contributor to RET) by rotenone following oxalate treatment caused a further increase in ROS levels (**Figure 2F**). These findings suggest RET may not be the primary source of oxalate induced ROS in macrophages.

### Oxalate Alters Mitochondrial Gene Expression in Macrophages

**Figure 3:**
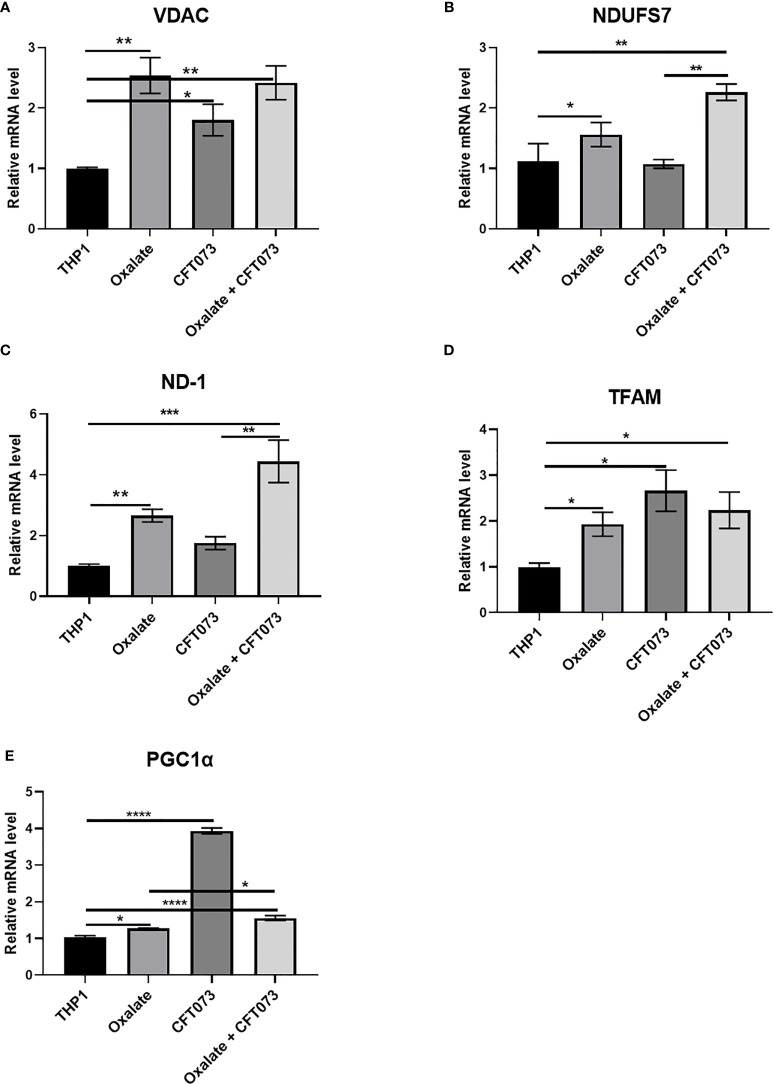
Oxalate increases the expression of key mitochondrial genes in THP-1 macrophages. THP-1 macrophages were exposed to oxalate with or without CFT073 (MOI 1:2) as described in the *Materials and Methods* section. mRNA levels of mitochondrial genes: **(A)** VDAC1 (voltage dependent anion channel 1), **(B)** NDUFS7 (NADH : Ubiquinone Oxidoreductase Core Subunit S7), **(C)** ND-1 (NADH dehydrogenase 1), **(D)** TFAM (Transcription Factor A, Mitochondrial), and **(E)** PGC1α (PPARG Coactivator 1 Alpha) were determined. All experiments were performed at least three times with n=5 replicates. Data are expressed as mean ± SEM. *p < 0.05, **p < 0.01, ***p < 0.001, ****p < 0.0001 is significant compared to control THP-1 macrophages or CFT073 alone.

To further evaluate the reason for compromised cellular bioenergetics following oxalate treatment, we evaluated gene expression of key mitochondrial genes critical for sustaining mitochondrial function. We determined that gene expression of VDAC1 (voltage dependent anion channel, a gene critical for maintaining cellular homeostasis) (41) was elevated following oxalate treatment. Likewise, levels of PGC1α (PPARG Coactivator 1 Alpha), an inducible transcription factor that regulates the metabolism, polarization, and activation of macrophages (42), were elevated in oxalate treated cells. Similarly, the expression of Transcription Factor A, Mitochondrial (TFAM; a regulator of mitochondrial biogenesis and function) (43) and mitochondrial Complex I subunits, NADH dehydrogenase 1 (ND1) and NADH : Ubiquinone Oxidoreductase Core Subunit S7 (NDUFS7) were significantly upregulated following oxalate exposure (**Figures 3A**–**E**). In addition, oxalate treated macrophages exposed to CFT073 had a further increase in expression for all of these genes except PGC1ɑ (**Figure 3**). CFT073 alone increased the expression of VDAC1, TFAM and PGC1α (**Figures 3A, D, E**). Notably, CFT073 had no effect on NDUFS7 or ND-1 (**Figures 3B, C**).

### Oxalate Exposed Macrophages Have Compromised Anti-Bacterial Response

**Figure 4:**
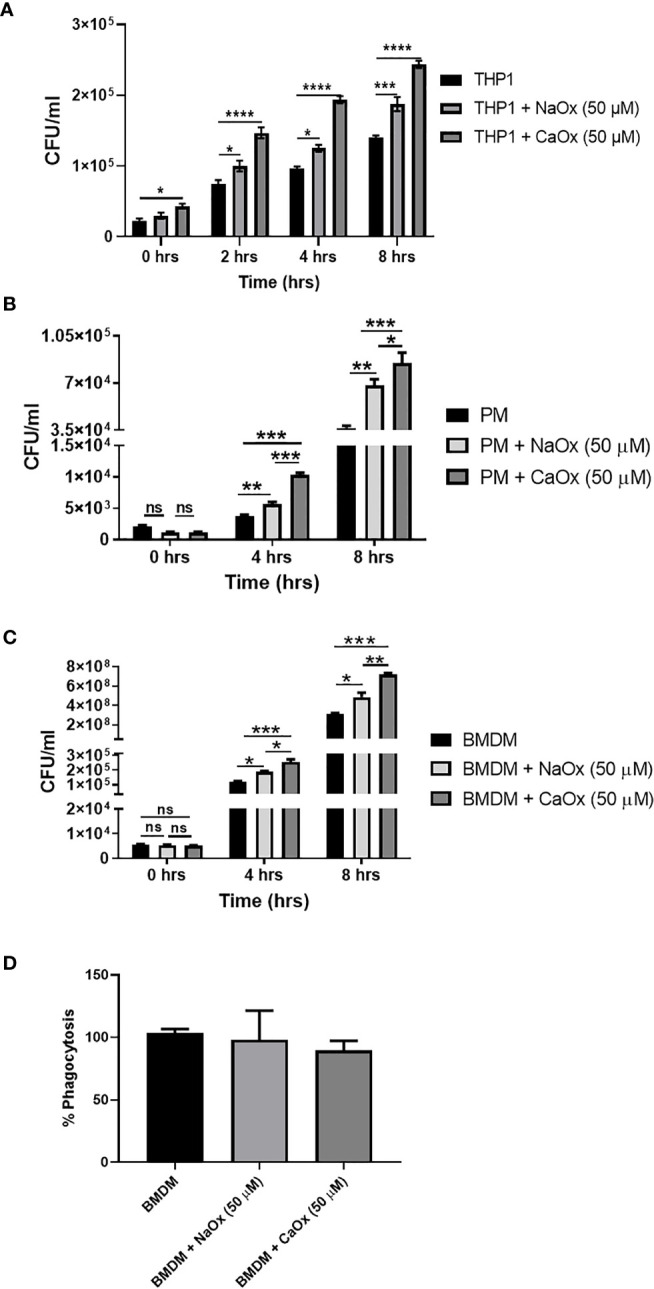
Oxalate increases bacterial burden in macrophages. Macrophages were exposed to sodium oxalate (NaOx; 50 μM) or calcium oxalate crystals (CaOx; 50 μM) for 72 hrs followed by CFT073 infection (MOI 1: 5) for 2, 4, or 8 hrs. Data represent CFU recovery from **(A)** THP-1 macrophages; **(B)** Peritoneal macrophages, and **(C)** BMDMs. **(D)** Percent phagocytosis of BMDM. All experiments were performed in triplicates. Data are expressed as mean ± SEM. ns, not significant *p < 0.05, **p < 0.01, ***p < 0.001, ****p < 0.0001 is significant compared to control macrophages.

To understand the functional relevance of oxalate on macrophages, we evaluated the antibacterial response of THP-1 macrophages treated with oxalate to CFT073. The intracellular bacterial burden significantly increased in a time-dependent manner in macrophages exposed to oxalate [i.e. NaOx (soluble form) and CaOx crystals (insoluble form)] compared to control cells (**Figure 4A**). The amount of extracellular bacteria in the cellular media from oxalate treated macrophages were also increased compared to control cells (**Supplementary Figures 1A, B**). We also evaluated whether the effects of oxalate on CFT073 occurs in primary macrophages. Using peritoneal macrophages and BMDM from mice, we showed that CFT073 permits a significantly higher bacterial recovery from oxalate treated peritoneal macrophages as well as BMDM (**Figures 4B, C**). Cells treated with CaOx also had increased microbial survival and growth inside macrophages. Interestingly, at T=0 (time immediately after establishment of infection), there was no significant difference in the bacterial burden across the groups suggesting the differences observed in CFU are not due to a differential uptake of CFT073 in primary macrophages. This assertion is further strengthened by the results of phagocytosis assays where we did not observe any significant differences in phagocytosis among the treatment groups (**Figure 4D**).

### Oxalate Increases Pro-Inflammatory Cytokine and Reduces Anti-Inflammatory Cytokine mRNA Levels and Secretion in Macrophages

**Figure 5:**
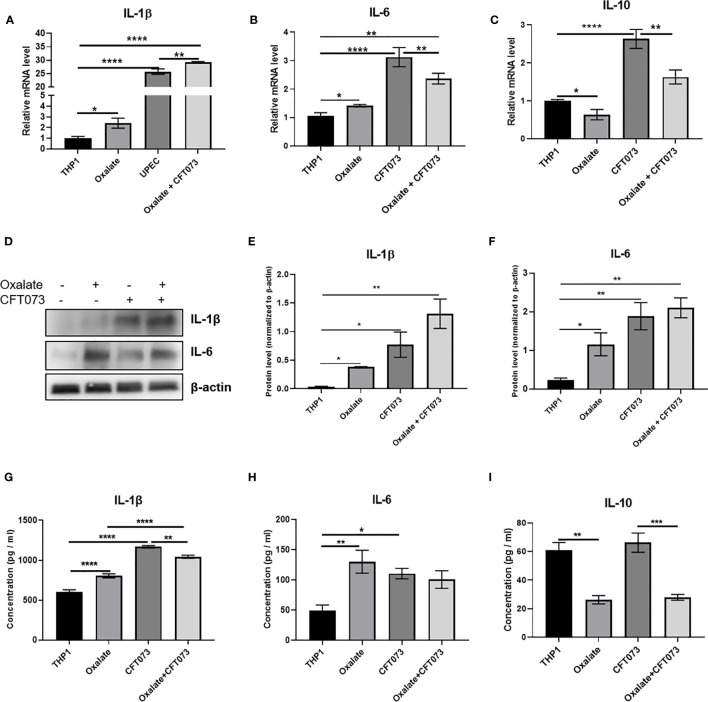
Oxalate increases pro-inflammatory cytokine expression and secretion but reduces anti-inflammatory cytokine expression and secretion in THP-1 macrophages. THP-1 macrophages were exposed to oxalate with or without CFT073 (MOI 1:2) as described in the *Materials and Methods* section. Gene expression of **(A)** IL-1β (pro-inflammatory cytokine), **(B)** IL-6 (pro-inflammatory cytokine), and **(C)** IL-10 (anti-inflammatory cytokine) using qRT-PCR. **(D)** Western blot analysis and **(E, F)** quantification of IL-6 and IL-1β levels using ImageJ software. Cytokine secretion of **(G)** IL-1β, **(H)** IL-6, and **(I)** IL-10, respectively. All experiments were performed at least three times with n=5 replicates. Data are expressed as mean ± SEM. *p < 0.05, **p < 0.01, ***p < 0.001, ****p < 0.0001 is significant compared to control THP-1 macrophages or CFT073 alone.

A highly oxidizing cellular environment can trigger inflammation. Macrophages exposed to oxalate had perturbed expression and secretion of cytokines involved in inflammation and immune response following CFT073 exposure, relative to control cells (**Figures 5A**–**I**). In particular, the mRNA levels of pro-inflammatory cytokines, IL-1β and IL-6, were significantly elevated in all treatment groups (**Figures 5A, B**). As shown in **Figure 5C**, oxalate significantly reduced the expression of the anti-inflammatory cytokine, IL-10. CFT073 treated macrophages had a significant increase in IL-10 mRNA levels compared to control THP-1 cells. However, macrophages exposed to oxalate plus CFT073 had a significant reduction in IL-10 expression compared to CFT073 treated cells (**Figure 5C**). Western blot analysis further supported the mRNA data and demonstrated increased IL-1β and IL-6 protein levels (**Figures 5D**–**F**). Lastly, IL-1β, IL-6, and IL-10 cytokine secretion were consistent with the observed effects of oxalate on mRNA and protein levels (**Figures 5G**–**I**).

## Discussion

Calcium oxalate stone formers can sometimes have recurrent UTI as well as stone recurrence (24, 25). Although the etiology of these two diseases are quite different, the immune system contributes to both pathologies and associations between UTI and kidney stones have been reported (14, 21–25). We sought to explore this potential association further by investigating the impact of oxalate on macrophages. Monocytes and macrophages have been proposed to be involved in CaOx stone disease (44–46). We previously reported that monocytes from CaOx kidney stone formers have compromised bioenergetic health (26). We further found that oxalate impairs metabolism and redox homeostasis in human primary monocytes and in an *in vitro* monocyte cell line (27). CaOx crystals have been reported to alter mitochondrial protein levels in human monocytes (47) and to promote their differentiation into pro-inflammatory macrophages (29). Based on investigations of others and the aforementioned findings (48), we hypothesized oxalate disrupts macrophage cellular bioenergetics and redox homeostasis, promotes inflammation, and this weakens their response to bacterial exposure.

We exposed monocytes to oxalate to mimic physiological conditions when monocytes are exposed to soluble oxalate *in vivo*. Once monocytes infiltrate the kidney and differentiate into macrophages, they are exposed to either soluble or insoluble (e.g. crystals) oxalate. It has been well established that increased oxalate concentrations in the kidney can result in crystal deposition (49). The THP-1 model has been shown to be useful to study the effects of dietary components on macrophage cell function (29, 50, 51) and the effects of different stimuli on the bacterial killing of macrophages (52, 53). Additionally, THP-1 cells have been used to study UTIs caused by UPEC induced cystitis, pyelonephritis, etc. (53–55). We determined that oxalate reduced cellular bioenergetics in macrophages and this was consistent with our previous findings where we observed oxalate suppressed cellular bioenergetics in monocytes (27). This suppression was more prominent when oxalate treated macrophages were exposed to the UPEC strain, CFT073. We investigated whether deficits in OCR and energy production could be compensated by a shift to glycolysis. However, we observed a reduction in glycolysis in the cells exposed to oxalate only. However, these deficits in glycolysis became more pronounced and significant in cells exposed to CFT073 alone or in the presence of oxalate. These data suggested that a decline in oxidative phosphorylation in cells treated with oxalate, CFT073 or in combination could not switch to the glycolytic pathway. Additionally, ATP levels were significantly decreased in macrophages exposed to oxalate with or without CFT073 exposure. These data further support the premise that glycolytic ATP production does not compensate for a reduced ATP generation *via* oxidative phosphorylation.

Cellular bioenergetics and ATP production during infection is a function of host-pathogen interaction wherein multiple factors including cellular micro-environment, redox and metabolic status, expression of key genes/pathways, cellular health etc. can profoundly impact ATP levels (48, 56–58). Majority of the cell’s ATP is provided by oxidative phosphorylation and movement of reducing equivalents like NADH through the electron transport chain within the inner mitochondrial membrane (59). The decrease in mitochondrial respiration following oxalate exposure as indicated by a reduction in OCR could be attributed to the significant decline in mitochondrial Complex I and IV activities observed in oxalate treated cells. This in turn could directly impact cellular bioenergetics and reduce macrophage energy production. This raises an important question about additional compensatory mechanisms needed to overcome the reduction in Complex I and Complex IV activity and ATP levels in oxalate treated macrophages. Indeed, we observed an increased expression of crucial mitochondrial genes such as VDAC1 that are known to regulate cellular metabolism and redox homeostasis (41). Likewise, the mitochondrial gene PGC1ɑ was induced with oxalate exposure and is known for regulating cellular processes such as cytokine signaling, metabolic polarization, and activation of macrophages (42). In addition, PGC1ɑ promotes mitochondrial biogenesis by activating the transcription factor, TFAM, which helps to maintain mitochondrial homeostasis during stress (43, 60). Induction of TFAM and PGC1α expression following oxalate exposure in macrophages suggests a compensatory response involving mitochondrial biogenesis. In accordance, we saw a significant upregulation of mitochondrial Complex I gene subunits, ND1 and NDUFS7 gene expression. Further, MitoTracker green was increased suggesting mitochondrial biogenesis was stimulated. Overall, our data show activation of mitochondrial biogenesis as a possible attempt to improve metabolism and energy production in macrophages in response to oxalate. The attempt of macrophages to improve cellular bioenergetics has been observed in other diseases impacting mitochondria (61–63). Nonetheless, this compensatory biological response is maladaptive, as it fails to sufficiently correct for the ATP deficit as observed in other health conditions (64).

The ability of macrophages to remove pathogens and crystals is highly dependent on redox homeostasis and immune signaling (47, 65). We determined that mitochondrial superoxide and total ROS levels were significantly elevated in oxalate treated macrophages with and without CFT073 treatment. Mitochondrial superoxide can be quickly dismutated to H_2_O_2_ by superoxide dismutase. Oxalate treated macrophages also displayed elevated levels of H_2_O_2_. Increased H_2_O_2_ can disturb redox homeostasis and cause extensive inflammation and cell damage (47, 65). In addition, we determined NADPH was depleted in all treatment groups, which further indicated disturbed redox homeostasis (66, 67). Mitochondrial membrane potential was also increased in macrophages following oxalate exposure. Increased mitochondrial membrane potential and H_2_O_2_ levels are potential indicators of RET (68, 69). In this study, rotenone exposure increased ROS generation which suggested that RET is not the exclusive source of ROS and forward electron transport mediated ROS production may be occurring in oxalate treated macrophages. Sufficient mechanistic details to evaluate forward electron transport and RET mediated ROS are still a matter of investigation (70). More in depth studies are needed to delineate the primary source of ROS in oxalate treated cells.

Lastly, we aimed to test the functional relevance of elevated ROS and compromised bioenergetic function towards the cell’s response to bacterial infection. Exposure to sodium oxalate reduced the anti-bacterial response of macrophages as shown by increased bacterial recovery. This bacterial recovery was further exacerbated when cells were exposed to CaOx crystals. These observations were also confirmed in primary peritoneal macrophages and BMDM from mice. Perhaps the morphology of the crystals was able to induce additional damage to the cells compared to the soluble form of oxalate, as crystals have been reported to cause significant injury to renal cells (71) and monocytes (27). What could be the cause for increased recovery and progression of infection following exposure with CFT073 in macrophages? There was no significant difference in bacterial uptake or phagocytosis in oxalate treated cells at the time of infection (T=0). Thus, this rules out the possibility that differences in microbial uptake are responsible for differences in CFU recovery. It is possible the elevated levels of ROS in oxalate treated cells could impact immune responses by modulating the secretion of cytokines that induce innate immunity (72). Mitochondrial ROS at complex I can trigger the transcription of IL-1β (pro-inflammatory cytokine). We determined that IL-1β and caspase-1, were stimulated by oxalate with or without CFT073. Our findings are consistent with earlier reports where pro-inflammatory cytokines such as IL-6 and IL-1β have been found to be upregulated in a CaOx mouse experimental model (73). In addition, oxalate inhibited the secretion of IL-10, an anti-inflammatory cytokine in macrophages. Disruption in IL-10 production is known to increase inflammatory activity and to impair macrophage metabolism, the clearance of defective macrophages and their phagocytic capacity (74, 75). Complex I activity may be critical during bacterial infection, since deficiency of Complex I subunits reduces bacterial clearance during infection (76). The impairment of Complex I and IV activity in oxalate treated macrophages may contribute to reduced macrophage clearance and increased inflammation.

Taken together, our data suggests elevated levels of oxidative stress and reduced cellular bioenergetics following oxalate exposure compromises the protective mechanisms of macrophages and results in increased bacterial burden. Further studies assessing the interaction between CaOx crystals and macrophages are needed to delineate additional mechanisms leading to disruption in mitochondrial and cellular homeostasis in macrophages.

## Data Availability Statement

The raw data supporting the conclusions of this article will be made available by the authors, without undue reservation.

## Ethics Statement

The animal study was reviewed and approved by The Institutional Animal Ethics Committee, All India Institute of Medical Sciences, New Delhi, India (Project id: N1125).

## Author Contributions

Designed the research: VS and TM. Performed experiments: PK, KS, VS, and TM. Analyzed data: PK, KS, VS, and TM. Wrote the manuscript: PK, KS, VS, and TM. All authors contributed to the article and approved the submitted version.

## Funding

This research was supported by: NIH grants DK106284 and DK123542 (TM) and DK079337 (UAB O’Brien Center); Oxalosis & Hyperoxaluria Foundation – American Society of Nephrology KidneyCure Transition to Independence Grant (TM); Intramural Research Grant AIIMS (A-638), HarGobind Khorana IYBA (BT/11/IYBA/2018/01), DST-SERB Core Grant (SERB/F/8989/2019-2020) and LSRB-375/SH&DD/2020 (VS).

## Conflict of Interest

The authors declare that the research was conducted in the absence of any commercial or financial relationships that could be construed as a potential conflict of interest.

## Publisher’s Note

All claims expressed in this article are solely those of the authors and do not necessarily represent those of their affiliated organizations, or those of the publisher, the editors and the reviewers. Any product that may be evaluated in this article, or claim that may be made by its manufacturer, is not guaranteed or endorsed by the publisher.
