# Supplementary material for: Oxalate Alters Cellular Bioenergetics, Redox Homeostasis, Antibacterial Response, and Immune Response in Macrophages
Source: Front Immunol. 2021 Oct 21;12:694865. doi: 10.3389/fimmu.2021.694865 (PMC8566947; doi:10.3389/fimmu.2021.694865)
Supplement: Supplementary file 1 [file Presentation_1.pptx]

## Slide 1
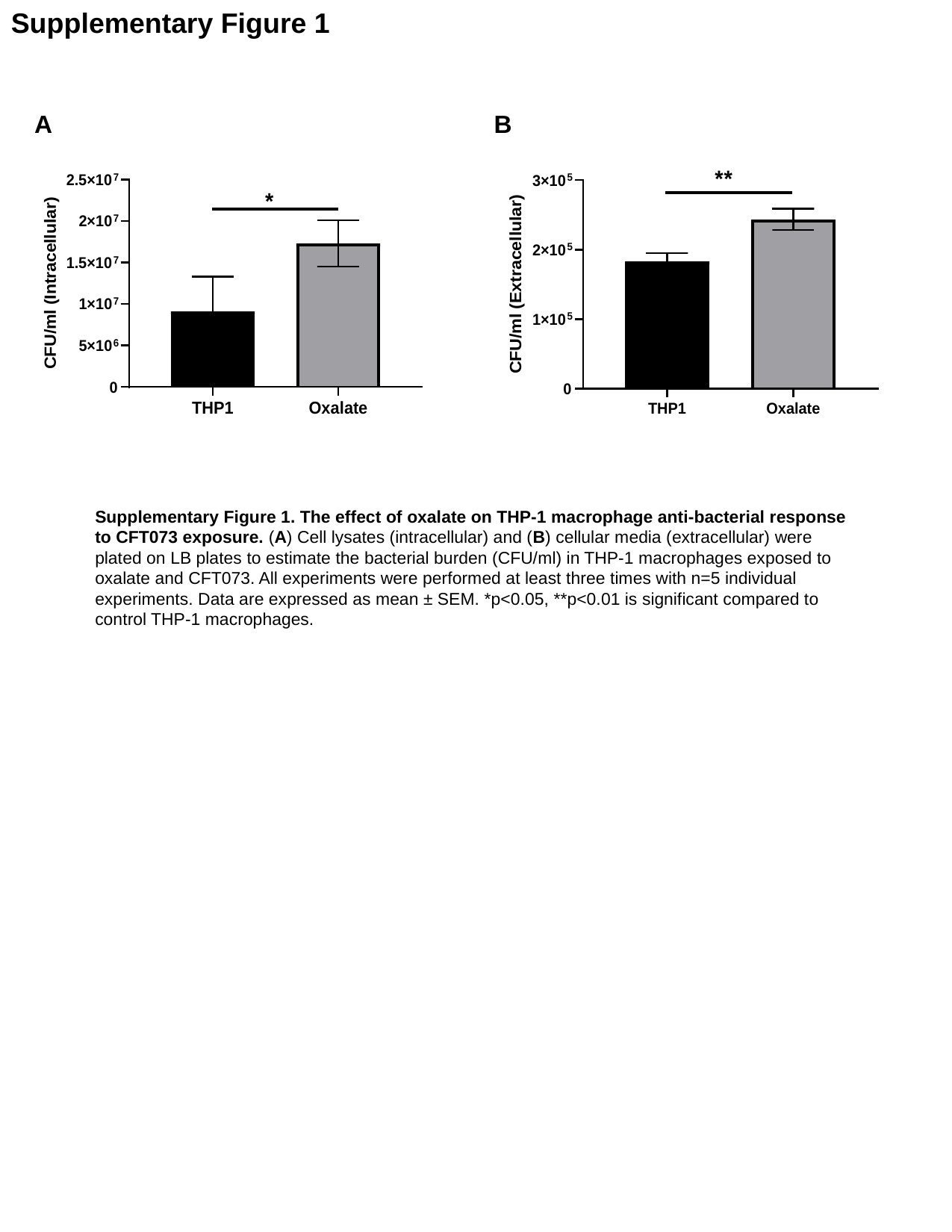

Supplementary Figure 1
A
B
Supplementary Figure 1. The effect of oxalate on THP-1 macrophage anti-bacterial response to CFT073 exposure. (A) Cell lysates (intracellular) and (B) cellular media (extracellular) were plated on LB plates to estimate the bacterial burden (CFU/ml) in THP-1 macrophages exposed to oxalate and CFT073. All experiments were performed at least three times with n=5 individual experiments. Data are expressed as mean ± SEM. *p<0.05, **p<0.01 is significant compared to control THP-1 macrophages.
